# Essential role of prostaglandin E_2_ and the EP3 receptor in lymphatic vessel development during zebrafish embryogenesis

**DOI:** 10.1038/s41598-019-44095-5

**Published:** 2019-05-21

**Authors:** Ryo Iwasaki, Kyoshiro Tsuge, Koichiro Kishimoto, Yuta Hayashi, Takuya Iwaana, Hirofumi Hohjoh, Tomoaki Inazumi, Atsuo Kawahara, Soken Tsuchiya, Yukihiko Sugimoto

**Affiliations:** 1 Department of Pharmaceutical Biochemistry, Graduate School of Pharmaceutical Sciences, Kumamoto University, 5-1 Oe-honmachi, Chuo-ku, 862-0973 Kumamoto, Japan; 2 Japan Agency for Medical Research and Development-Core Research for Evolutional Science and Technology (AMED-CREST), 1-7-1 Otemachi, Chiyoda-ku, 100-0004 Tokyo, Japan; 3 Laboratory for Developmental Biology, Center for Medical Education and Sciences, Graduate School of Medical Science, University of Yamanashi, 1110 Shimokato, Chuo, 409-3898 Yamanashi, Japan

**Keywords:** Cell lineage, Lymphangiogenesis

## Abstract

Lymphatic endothelial cells arise from the venous endothelial cells in embryonic lymphatic development. However, the molecular mechanisms remain to be elucidated. We here report that prostaglandin (PG) E_2_ plays essential roles in the embryonic lymphatic development through the EP3 receptor, one of the PGE_2_ receptors. Knockdown of the EP3 receptor or inhibition of cyclooxygenases (COX; rate-limiting enzymes for PG synthesis) impaired lymphatic development by perturbing lymphatic specification during zebrafish development. These impairments by COX inhibition were recovered by treatment with sulprostone (EP1/3 agonist). Knockdown of the EP3 receptor further demonstrated its requirement in the expression of sex determining region Y-box 18 (sox18) and nuclear receptor subfamily 2, group F, member 2 (nr2f2), essential factors of the lymphatic specification. The EP3 receptor was expressed in the posterior cardinal vein (region of embryonic lymphatic development) and the adjacent intermediate cell mass (ICM) during the lymphatic specification. COX1 was expressed in the region more upstream of the posterior cardinal vein relative to the EP3 receptor, and the COX1-selective inhibitor impaired the lymphatic specification. On the other hand, two COX2 subtypes did not show distinct sites of expression around the region of expression of the EP3 receptor. Finally, we generated EP3-deficient zebrafish, which also showed defect in lymphatic specification and development. Thus, we demonstrated that COX1-derived PGE_2_-EP3 pathway is required for embryonic lymphatic development by upregulating the expression of key factors for the lymphatic specification.

## Introduction

The lymphatic system is a major component of the vertebrate vasculature and plays pivotal roles in the collection of interstitial fluid, absorbance of dietary lipids, and trafficking of immune cells^1^. Development of the lymphatic system begins in the early developmental stages, and lymphatic endothelial cells develop from endothelial cells in the posterior cardinal vein^2,3^. Vascular endothelial growth factor c (vegfc) and its receptor, fms-related tyrosine kinase 4 (flt4; also known as vascular endothelial growth factor receptor 3, vegfr3) are key factors for lymphatic specification^4–8^. Vegfc released by the dorsal aorta induces the lymphatic specification through binding to flt4 expressed in venous endothelial cells. Sex determining region Y-box 18 (sox18), a member of sox transcription factor family, is also essential for the lymphatic specification through the induction of transcriptional factors, such as nuclear receptor subfamily 2, group F, member 2 (nr2f2; also known as COUP transcription factor 2, COUP-TFII)^8–10^. Nr2f2 controls the expression of lymphatic genes, such as lymphatic vessel endothelial hyaluronic receptor 1b (lyve1b; a lymphatic marker)^11–13^. Additionally, apelin (apln), collagen and calcium binding EGF domains 1 (ccbe1), and wingless-type MMTV integration site family, member 5b (wnt5b) (secreted proteins) were reported to promote lymphatic development^6,14–16^. On the other hand, bone morphogenetic protein 2b (bmp2b) was shown to negatively modulate the development of lymphatic endothelial cells^17^.

Prostaglandin (PG) E_2_ is an arachidonate metabolite that is synthesized via a pathway with cyclooxygenase (COX) as the rate-limiting enzyme. PGE_2_ has been shown to exert a variety of actions by binding to four specific G-protein-coupled receptors (EP1, EP2, EP3, and EP4) on the plasma membrane of neighboring cells in humans and mice^18,19^. The EP1 receptor is coupled to Gq to increase intracellular Ca^2+^ concentrations. The EP2 and EP4 receptors are coupled to Gs and increase intracellular cAMP concentrations via the activation of adenylyl cyclase. The EP3 receptor is coupled to Gi, and mediates the inhibition of adenylyl cyclase and the upregulation of intracellular Ca^2+^ concentrations^20,21^. The formation of lymphatic vessels is known to occur during not only embryogenesis but also the progression of various cancers^22^, and COX2-derived PGE_2_ was found to facilitate lymphangiogenesis during tumor development through the EP3 receptor in mice^23,24^. In addition, PGE_2_ was shown to accelerate the formation of lymphatic vessels through the EP3 receptor in granulation tissues^25,26^. These data suggest the possibility that the PGE_2_-EP3 pathway plays important roles in the formation of lymphatic vessels. However, it remains unknown whether the PGE_2_-EP3 pathway is involved in the development of lymphatic vessels during embryogenesis.

To identify the role of the PGE_2_-EP3 pathway in lymphatic vessel development during embryogenesis, we used zebrafish as a model organism, because zebrafish embryos are optically clear and undergo rapid early development outside the maternal body^27^. In addition, zebrafish share many similarities in their molecular mechanisms of lymphatic vessel formation with other vertebrates, and express three COX subtypes (COX1, COX2a, and COX2b) and eight PGE_2_ receptor subtypes (EP1a, EP1b, EP2a, EP2b, EP3, EP4a, EP4b, and EP4c)^28–31^. Here, we report novel functions of the PGE_2_-EP3 pathway in the formation of lymphatic vessels during early development.

## Results

### The PGE_2_-EP3 pathway is involved in lymphatic vessel formation during early development

**Figure 1:**
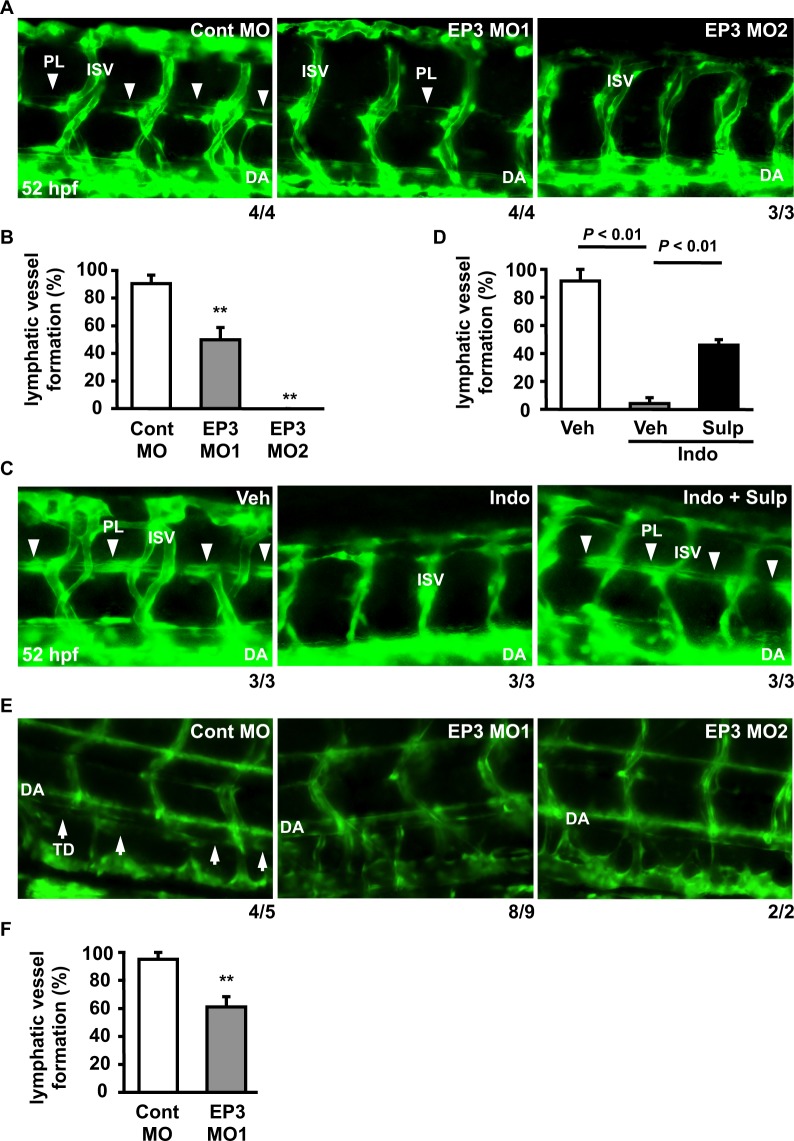
Role of the PGE_2_-EP3 pathway in lymphatic vessel formation. (**A**) Tg(fli1a:egfp) embryos were injected with Cont MO, EP3 MO1, or MO2. Images of the trunks were taken at 52 hpf. (**B**) The ratio of PL-positive segments in nine consecutive segments of (**A**) was quantitated. Each value represents the mean ± SEM (N = 3–4). (**C**) Tg(fli1a:egfp) embryos were treated with vehicle (Veh) or indomethacin (Indo; 100 μM) in the absence or presence of sulprostone (Sulp; 1 μM) from 0 to 52 hpf. Images were taken at 52 hpf. (**D**) The ratio of PL-positive segments in nine consecutive segments of (**C**) was quantitated. Each value represents the mean ± SEM (N = 3). (**E**) Tg(fli1a:egfp) embryos were injected with Cont MO, EP3 MO1, or EP3 MO2. Images of the trunks were taken at 5 dpf. (**F**) The ratio of TD-positive segments in eight consecutive segments of Cont MO- or EP3 MO1-injected morphants was quantitated. Each value represents the mean ± SEM (N = 5–9). **P < 0.01 vs Cont MO. DA: dorsal aorta; ISV: intersomitic vessel; PL: parachordal lymphangioblast; TD: thoracic duct. The PL is indicated by arrowheads, and the TD is indicated by arrows. The number at the bottom right of each panel indicates the number of embryos demonstrating the phenotype shown in the panel over the total number of embryos analyzed in a representative experiment.

To investigate whether the PGE_2_-EP3 pathway regulates lymphatic vessel formation in embryogenesis, we analyzed the effects of EP3 receptor knockdown on zebrafish lymphatic development using Tg(fli1a:egfp) embryos, in which both blood and lymphatic vessels are labeled^27,32^. We used two splice-blocking morpholino antisense oligos (MOs), EP3 MO1 and MO2 (Supplementary Fig. S1A, Supplementary Table S1). Quantitative analysis showed that injection of EP3 MO1 or MO2 both markedly decreased the mature mRNA expression level of the EP3 receptor at 24 hours post fertilization (hpf) (Supplementary Fig. S1B). To evaluate lymphatic vessel formation, we analyzed the parachordal lymphangioblast (PL) at the horizontal myoseptum, which is commonly used to study lymphatic development in zebrafish^9,27^. In control (Cont) MO-injected embryos, the PL was fully formed in most of the segments at 52 hpf (Fig. 1A,B). On the other hand, PL formation was severely impaired in morphants injected with EP3 MO1 or MO2 (Fig. 1A), and the ratio of PL-positive segments was also significantly reduced in these morphants (Fig. 1B). We then investigated whether indomethacin, a non-selective COX inhibitor^28^, induces lymphatic vessel defects. In vehicle-treated embryos, PL was normally formed in the horizontal myoseptum at 52 hpf. On the other hand, treatment with indomethacin inhibited PL formation and significantly reduced the ratio of PL-positive segments (Fig. 1C,D). This effect of indomethacin was substantially recovered by cotreatment with sulprostone, an EP1/3 agonist^30^ (Fig. 1C,D). Furthermore, we investigated the formation of thoracic duct (TD), which is located immediately ventral to the dorsal aorta in the trunk, at 5 days post fertilization (dpf) (Fig. 1E,F). In Cont MO-injected embryos, the TD was fully formed in most of the segments. On the other hand, TD formation was severely impaired in morphants injected with EP3 MO1 and MO2 (Fig. 1E), and the ratio of TD-positive segments was significantly reduced in the EP3 MO1-injected morphants (Fig. 1F). These results indicated that the PGE_2_-EP3 pathway plays an important role in lymphatic vessel formation during early development.

### The PGE_2_-EP3 pathway is required for lymphatic specification from venous to lymphatic endothelial cells

**Figure 2:**
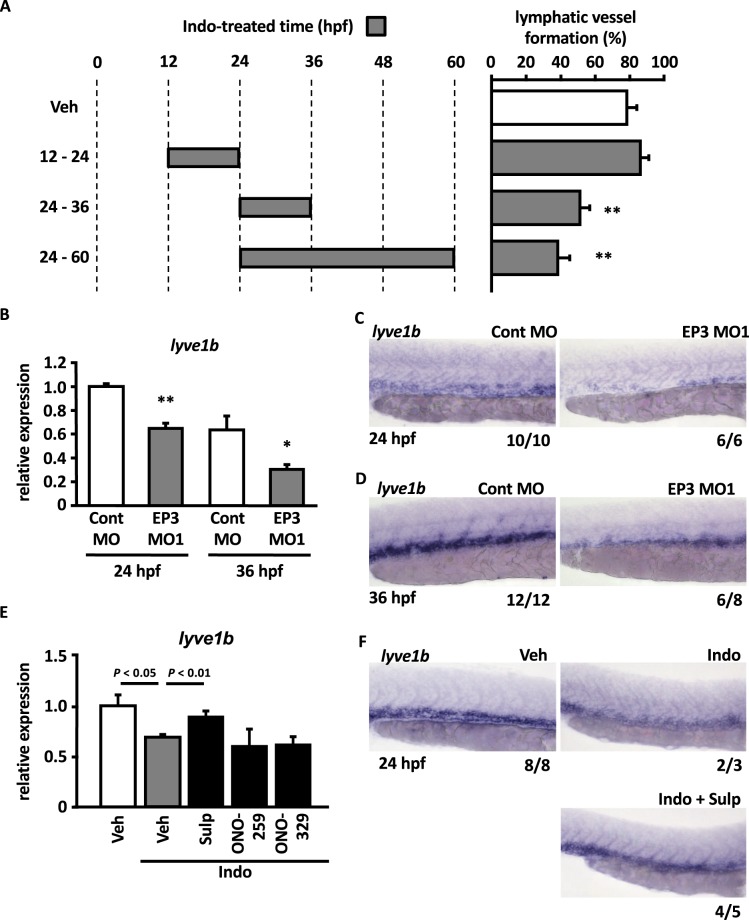
Role of the PGE_2_-EP3 pathway in the lymphatic specification from venous to lymphatic endothelial cells. (**A**) Tg(fli1a:egfp) embryos were treated with Veh or Indo (100 μM) for the indicated times. The ratio of PL-positive segments at 60 hpf was quantified. Each value represents the mean ± SEM (N = 5). **P < 0.01 vs Veh. (**B**) Relative expression levels of *lyve1b* were quantified by RT-qPCR in morphants at 24 and 36 hpf. Values are shown relative to the value obtained with Cont MO at 24 hpf. Each value represents the mean ± SEM (N = 3–4) *P < 0.05, **P < 0.01 vs Cont MO at each corresponding time. (C,D) Expression of *lyve1b* was analyzed by WISH in morphants at 24 hpf (**C**) and 36 hpf (**D**). (**E**,**F**) Zebrafish embryos were treated with Veh or Indo (100 μM) in the absence or presence of EP agonists (10 μM) from 0 to 24 hpf. The expression level of *lyve1b* was quantified by RT-qPCR (**E**). The values are shown relative to the value obtained with Veh. Each value represents the mean ± SEM (N = 3–4). Expression of *lyve1b* was analyzed by WISH at 24 hpf (**F**). The number at the bottom right of each panel indicates the number of embryos demonstrating the phenotype shown in the panel over the total number of embryos analyzed in a representative experiment.

Lymphatic endothelial cells are generated from pre-existing endothelial cells of the posterior cardinal vein from approximately 24 to 36 hpf, and subsequently sprout from the posterior cardinal vein after 36 hpf and migrate to colonize embryonic tissues. To identify the lymphatic development process in which the PGE_2_-EP3 pathway is involved, we performed time-dependent inhibition of PG synthesis by treatment with indomethacin for a limited time (Fig. 2A). There was no significant difference in the ratios of PL-positive segments between vehicle treatment and indomethacin treatment from 12 to 24 hpf, when the posterior cardinal vein develops. On the other hand, indomethacin treatment from 24 to 36 hpf significantly reduced the ratio of PL-positive segments. The degree of reduction by the treatment from 24 to 36 hpf was equivalent to that by the treatment from 24 to 60 hpf. These data suggest the importance of the PGE_2_-EP3 pathway in the lymphatic specification process. Therefore, to determine whether the PGE_2_-EP3 pathway acts during the first steps of lymphatic development, we analyzed the expression levels of *lyve1b*, which is a lymphatic marker^13^. Compared with control morphants, EP3 receptor morphants had significantly lower expression levels of *lyve1b* at both 24 and 36 hpf (Fig. 2B). Whole-mount *in situ* hybridization (WISH) analysis demonstrated that *lyve1b* was expressed around the posterior cardinal vein in Cont MO-injected embryos at both 24 and 36 hpf (Fig. 2C,D). By contrast, embryos injected with EP3 MO1 showed substantial decreases in *lyve1b*-derived signals around the posterior cardinal vein at both time points (Fig. 2C,D). Embryos treated with indomethacin also showed decreased expression levels of *lyve1b* at 24 hpf (Fig. 2E). This effect of indomethacin was significantly recovered by cotreatment with sulprostone but not ONO-AE1-259 (an EP2 agonist)^31^ or ONO-AE1-329 (an EP4 agonist)^31^ (Fig. 2E). WISH analysis of embryos at 24 hpf demonstrated that indomethacin markedly reduced *lyve1b*-derived signals around the posterior cardinal vein, where *lyve1b*-derived signals were observed in vehicle-treated embryos (Fig. 2F). *Lyve1b*-derived signals that were decreased by indomethacin were recovered to the levels similar to that of vehicle-treated embryos by the cotreatment of sulprostone (Fig. 2F). These results indicated that the PGE_2_-EP3 pathway contributes to the formation of lymphatic vessels by regulating the lymphatic specification, which is the first step of lymphatic development during embryogenesis.

### Expression levels of genes involved in the lymphatic specification

**Figure 3:**
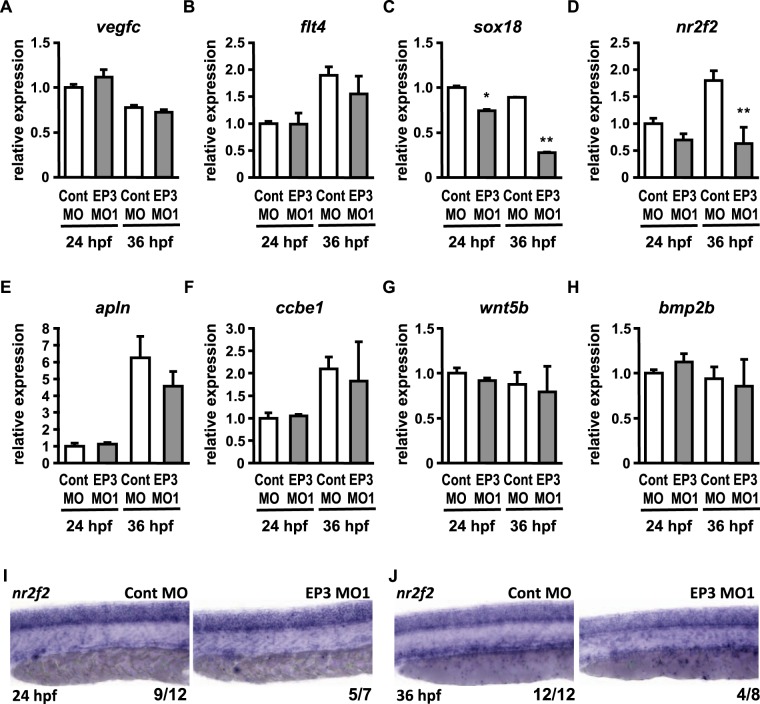
Expression of genes involved in lymphatic specification. (**A**–**H**) Relative expression levels (at 24 and 36 hpf) of genes involved in lymphatic specification were quantified by RT-qPCR in morphants injected with Cont MO or EP3 MO1. Values are shown relative to the value obtained with Cont MO at 24 hpf. Each value represents the mean ± SEM (N = 3). *P < 0.05, **P < 0.01 vs Cont MO at each corresponding time. (**I**,**J**) Expression of *nr2f2* was analyzed by WISH in morphants at 24 and 36 hpf. The number at the bottom right of each panel indicates the number of embryos demonstrating the phenotype shown in the panel over the total number of embryos analyzed in a representative experiment.

To analyze the function of the PGE_2_-EP3 pathway in the lymphatic specification, we investigated the mRNA expression level of various genes (*vegfc*, *flt4*, *sox18*, *nr2f2*, *apln*, *ccbe1*, *wnt5b*, and *bmp2b*) crucially involved in the lymphatic specification^4–10,14–17^. By reverse transcription-quantitative PCR (RT-qPCR), we analyzed the expression levels of these genes in EP3 receptor morphants at 24 and 36 hpf (Fig. 3A–H), which are early and late phases of lymphatic specification, respectively. At both 24 and 36 hpf, expression levels of *sox18* were significantly decreased in EP3 receptor morphants compared with control morphants (Fig. 3C). Expression levels of *nr2f2* at 36 hpf were also significantly decreased in EP3 receptor morphants, although expression levels of *nr2f2* at 24 hpf did not change (Fig. 3D). There was no significant difference in the expression levels of *vegfc*, *flt4* (also known as a vein marker at 24 hpf), *apln*, *ccbe1*, *wnt5b*, and *bmp2b* (Fig. 3A,B,E–H). Because *nr2f2* is expressed in not only the posterior cardinal vein but also the cranial and spinal cord^10^, we then examined the expression of *nr2f2* around the posterior cardinal vein at 24 and 36 hpf by WISH analysis (Fig. 3I,J). Signals of *nr2f2* were detected in the posterior cardinal vein and spinal cord in the trunk of Cont MO-injected embryos. In the trunk of EP3 MO1-injected embryos, *nr2f2-derived* signals were decreased only in the posterior cardinal vein but not the spinal cord, specifically in 36 hpf but not 24 hpf. These data indicated that the EP3 receptor plays important roles in the expression of *sox18* and *nr2f2* in the lymphatic specification.

### COX1-derived PGE_2_ regulates the lymphatic specification through the EP3 receptor

**Figure 4:**
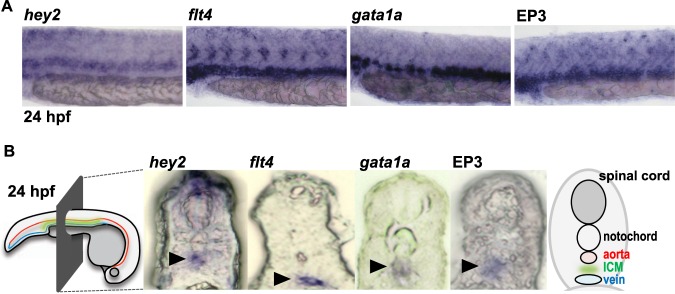
EP3 receptor mRNA is expressed in the posterior cardinal vein and the neighboring ICM. (**A**,**B**) Regions of expression of marker genes and the EP3 receptor were analyzed by WISH at 24 hpf (**A**) and subsequently by microscopic examination of their cross-sections (**B**). Arrowheads indicate the regions where the signals derived from each gene were observed.

We performed WISH analysis to identify the sites of EP3 receptor expression during the lymphatic specification. As previously reported^33^, EP3 receptor-derived signals were detected around blood vessels of the trunk at 24 hpf (Fig. 4A). To analyze these expression sites in more detail, we compared the signals of the EP3 receptor with those of several tissue markers at 24 hpf, namely, hes-related family bHLH transcription factor with YRPW motif 2 (hey2; an aorta marker)^34^, *flt4* (a vein marker)^35^ and GATA binding protein 1a (gata1a; an erythrocyte progenitor marker)^36^, which is reported to be exclusively expressed in the intermediate cell mass (ICM) between the dorsal aorta and the posterior cardinal vein at this developmental stage^36^. The signals of the EP3 receptor were located ventral to those of *hey2* and around those of *flt4* and *gata1a* (Fig. 4A). Furthermore, we made transverse sections of these embryos that were subjected to WISH analysis (Fig. 4B). As previously reported^34^, *hey2*-derived signals were detected under the notochord in transverse sections. Signals of *flt4* were located in a more ventral region than signals of *hey2*, and signals of *gata1a* were observed in a region between those of *hey2* and *flt4*. EP3 receptor-derived signals appeared to be located in the region with both *flt4-*derived and *gata1a*-derived signals. These results indicated that the EP3 receptor is expressed in the ICM and the posterior cardinal vein, where lymphatic endothelial cells are generated.

**Figure 5:**
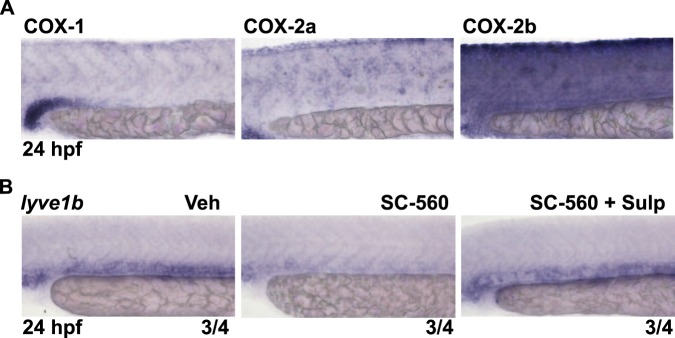
COX1-derived PGE_2_ is involved in the lymphatic specification. (**A**) Expression of COX1, COX2a, and COX2b was analyzed by WISH at 24 hpf. (**B**) Zebrafish embryos were treated with Veh or SC-560 (25 μM) in the absence or presence of Sulp (10 μM) from 0 to 24 hpf, and the expression of *lyve1b* was analyzed by WISH at 24 hpf. The number at the bottom right of each panel indicates the number of embryos demonstrating the phenotype shown in the panel over the total number of embryos analyzed in a representative experiment.

COX are rate-limiting enzymes for the biosynthesis of PGE_2_. As zebrafish have three subtypes of COX (COX1, COX2a, and COX2b), we tried to identify the specific COX subtypes involved in the lymphatic specification. We first performed WISH analysis at 24 hpf to identify the region of expression of each COX subtype during the lymphatic specification. COX1-derived signals were detected in the region more upstream of the posterior cardinal vein relative to EP3 receptor-derived signals (Fig. 5A). On the other hand, COX2a- and COX2b-derived distinctive signals were not detected in the trunk of zebrafish embryos (Fig. 5A). To clarify whether COX1 is involved in the lymphatic specification, we treated zebrafish embryos with a COX1-selective inhibitor, SC-560^37^ and investigated the expression levels of *lyve1b* at 24 hpf using WISH analysis. SC-560 diminished *lyve1b*-derived signals around the posterior cardinal vein (Fig. 5B). The decrease in *lyve1b*-derived signals was recovered by cotreatment with sulprostone (Fig. 5B). These results suggested that COX1-derived PGE_2_ plays important roles in the lymphatic specification from venous to lymphatic endothelial cells during early development.

### Impairment of lymphatic specification and development by EP3 receptor deficiency

**Figure 6:**
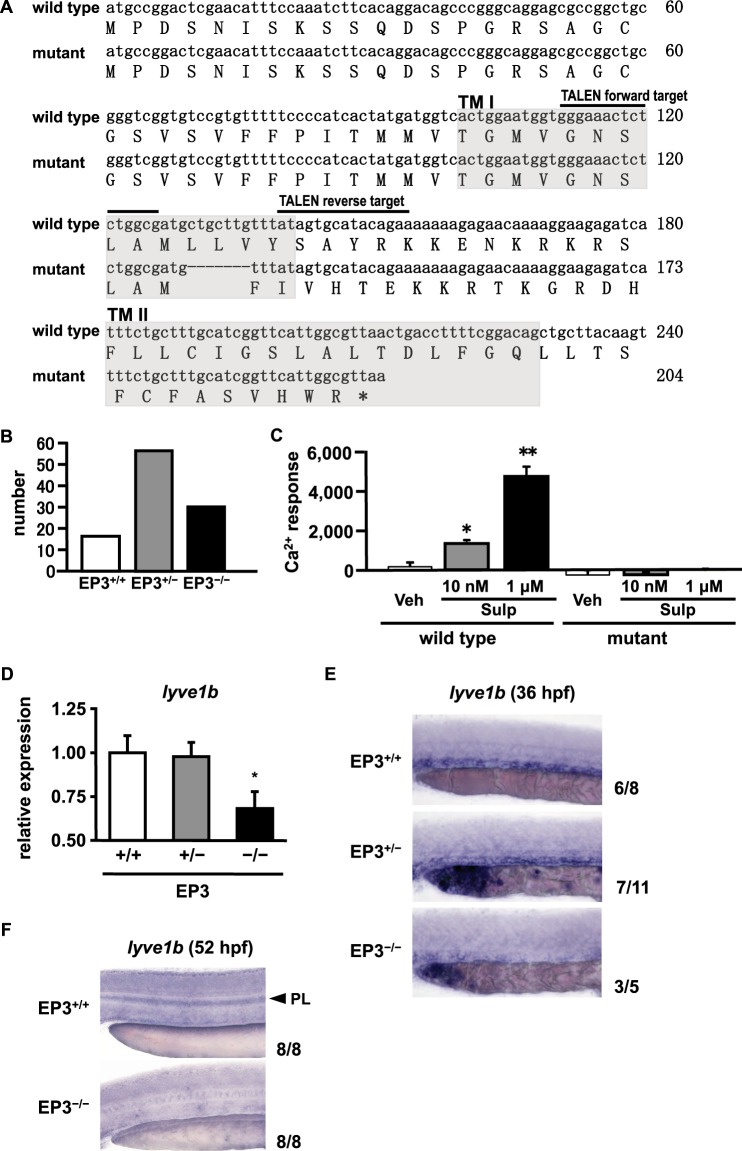
Effect of EP3 receptor deficiency on lymphatic specification and development. (**A**) Sequence alignment of the wild-type and mutant EP3 receptor. The seven base-pair deletion in mutant EP3 receptor is indicated by a dotted line. Transmembrane (TM) regions of the wild-type EP3 receptor are indicated by gray boxes. (**B**) Number of live births of EP3^+/+^, EP3^+/−^, and EP3^−/−^ zebrafish. (**C**) HeLa cells were transfected with an expression vector encoding either the wild-type or mutant EP3 receptor. After 24 h, cells were incubated with loading buffer for 1 h and then treated with Veh or Sulp. Induced intracellular Ca^2+^ mobilization was evaluated by AUC analysis. *P < 0.05, **P < 0.01 vs Veh. Each value represents the mean ± SEM (N = 3). (**D**) Relative expression levels of *lyve1b* were quantified by RT-qPCR in EP3^+/+^, EP3^+/−^, and EP3^−/−^ zebrafishs at 24 hpf. Values are shown relative to the value obtained with EP3^+/+^. Each value represents the mean ± SEM (N = 8–12) *P < 0.05 vs EP3^+/+^. (**E**) Expression of *lyve1b* was analyzed by WISH in EP3^+/+^, EP3^+/−^, and EP3^−/−^ zebrafish at 36 hpf. (**F**) Expression of *lyve1b* was analyzed by WISH in EP3^+/+^ and EP3^−/−^ zebrafish at 52 hpf. The PL is indicated by an arrowhead. The number at the bottom right of each panel indicates the number of embryos demonstrating the phenotype shown in the panel over the total number of embryos analyzed in a representative experiment.

To confirm the role of the EP3 receptor in the lymphatic specification, we finally generated EP3 receptor-deficient (EP3^−/−^) zebrafish using transcription activator-like effector nucleases (TALEN)^38^. Sequencing analysis showed that a stop codon was introduced into the transmembrane II region of the wild-type EP3 receptor by the deletion of seven base-pairs in transmembrane I (Fig. 6A). EP3^+/−^ and EP3^−/−^ zebrafish were born at approximately expected Mendelian ratio and were viable (Fig. 6B). We then performed the Ca^2+^ mobilization assay to confirm whether this deletion actually results in a loss of the function of the EP3 receptor. Although HeLa cells transfected with a construct encoding the wild-type EP3 receptor dose-dependently induced Ca^2+^ mobilization by sulprostone, HeLa cells transfected with a construct encoding the mutant EP3 receptor did not respond to sulprostone (Fig. 6C). Subsequently, we investigated the expression levels of *lyve1b* in EP3^+/−^ and EP3^−/−^ zebrafish to evaluate the effects of EP3 receptor deficiency on the lymphatic specification. Compared with EP3^+/+^ zebrafish, EP3^−/−^ zebrafish but not EP3^+/−^ zebrafish had significantly lower expression levels of *lyve1b* at 24 hpf (Fig. 6D). Additionally, WISH analysis showed that the expression levels of *lyve1b* around the posterior cardinal vein were remarkably decreased in EP3^−/−^ zebrafish but not EP3^+/−^ zebrafish at 36 hpf (Fig. 6E). Finally, we investigated the expression of *lyve1b* at 52 hpf using WISH analysis to analyze PL formation in EP3^−/−^ zebrafish (Fig. 6F). In EP3^+/+^ zebrafish, *lyve1b* signals were detected in the horizontal myoseptum, indicating adequate PL formation. On the other hand, signals of *lyve1b* were not detected in EP3^−/−^ zebrafish, indicating failure of PL formation. These results indicated the importance of EP3 receptor expression in lymphatic specification and development. Thus, the defects of lymphatic specification and development observed upon EP3 receptor knockdown and inhibition of PG synthesis were also observed in EP3 receptor gene-deficient mutants.

## Discussion

In this study, we showed for the first time that the PGE_2_-EP3 pathway plays an essential role in embryonic lymphatic development, by regulating the lymphatic specification, which is the first step of embryonic lymphatic development (Figs 1, 2, 5, 6). We found that the EP3 receptor is important for the expression of *sox18* and *nr2f2* but not for *vegfc*, *flt4*, *apln*, *ccbe1*, *wnt5b*, and *bmp2b* (Fig. 3). The EP3 receptor was expressed in the posterior cardinal vein and the neighboring ICM (Fig. 4). On the other hand, COX1, but not two COX2 subtypes, was expressed in the region more upstream of the posterior cardinal vein relative to the region of expression of the EP3 receptor, and contributed to lymphatic specification (Fig. 5). Lymphatic vessels develop from endothelial cells in the posterior cardinal vein. Therefore, normal development of the posterior cardinal vein is important for lymphatic vessel development^2,3^. Our data using RT-qPCR and WISH analyses indicated that the expression levels of *flt4*, used as a maturation marker of venous endothelial cells, were not altered in EP3 receptor morphants at 24 hpf, when the posterior cardinal vein is fully developed (Fig. 3 and data not shown). Our observation in Tg(fli1a:egfp) zebrafish also showed that the posterior cardinal vein of EP3 receptor morphants was similar to that of control morphants at both 24 and 36 hpf (data not shown). Additionally, lymphatic vessel development was not impaired by the inhibition of PG synthesis from 12 to 24 hpf, when the posterior cardinal vein develops (Fig. 2A). These data indicated that the EP3 receptor does not affect the development of PCV, at least on evaluation of lymphatic development. However, further detailed studies are needed to precisely determine whether the PGE_2_-EP3 pathway is involved in the differentiation and/or maturation of the posterior cardinal vein, at a level at which there is no effect on lymphatic development.

We found that the EP3 receptor plays important roles in the expression levels of *sox18* and *nr2f2* (Fig. 3). These genes are transcriptional factors that facilitate the lymphatic specification^8–10^. It was reported that sox18 genetically interacts with vegfc in the early phase of lymphatic development^9^, and that sox18 is required for the expression of *nr2f2* in zebrafish at 24 hpf^11^. Therefore, most of the effects of the EP3 receptor on the lymphatic specification might be exerted by the regulation of *sox18* expression. These two transcriptional factors are expressed in the posterior cardinal vein^10,39^, where embryonic lymphatic development begins. Interestingly, EP3 receptors were found to be expressed in the posterior cardinal vein and the neighboring ICM (Fig. 4). These results suggested that the functions of the EP3 receptor in lymphatic specification were exerted directly (in the posterior cardinal vein) or indirectly (by certain secreted or plasma membrane-associated factors supplied from the adjacent ICM through a paracrine or juxtacrine route). Then, we investigated *in vitro* whether the EP3 receptor expressed in venous endothelial cells accelerates differentiation toward lymphatic endothelial cells as a direct consequence of endothelial cell-autonomous activation of the EP3 receptor. However, stimulation by the selective human EP3 receptor agonist ONO-AE-248 did not upregulate the expression levels of the lymphatic marker *LYVE1* in human umbilical vein endothelial cells (HUVECs), even when the human EP3 receptor was overexpressed in HUVECs (data not shown). On the other hand, expression levels of secreted regulatory factors such as *apln*, *ccbe1*, *wnt5b*, and *bmp2b* were not affected by knockdown of the EP3 receptor at both 24 and 36 hpf (Fig. 3). Additionally, there have been no reports to our knowledge regarding plasma membrane-localized molecules that are involved in the lymphatic specification. Further studies are required to fully understand the molecular mechanism of the lymphatic specification promoted by the PGE_2_-EP3 pathway.

In this study, we found that COX1-derived PGE_2_ accelerated the lymphatic specification during embryonic lymphatic development (Fig. 5), and the EP3 receptor had no effect on the expression levels of *vegfc* and *flt4* (Fig. 3). In contrast to our study, COX2-derived PGE_2_ was reported to accelerate lymphangiogenesis through the EP3 receptor in tumor implantation and granulation formation models^23,25,26^. Mice and human cells were used in these models and PGE_2_ upregulated the expression of *Vegfc* and *Flt4* through the EP3 receptor. Although the reason for these differences is unclear, a likely explanation is that these discrepancies may be owing to differences of venous endothelial cell types among species and tissues, and differences between embryos and adults. Additionally, the different results might be explained by the absence or presence of inflammation. In tumor implantation and granulation formation models, inflammation was induced and immune cells invaded the tissues. It has been reported that migrated macrophages produce Vegfc through the PGE_2_-EP3 pathway in granulation formation models^25,26^. On the other hand, there have been no reports stating that immune cells are involved in embryonic lymphatic development, particularly in lymphatic specification. Sox18 was reported to be critical for tumor-induced lymphangiogenesis^40^. It is therefore possible that the PGE_2_-EP3 pathway might promote lymphangiogenesis in tumors and granulation tissues by the upregulation of not only *Vegfc* and *Flt4* expression but also *Sox18* expression.

In summary, we found that the PGE_2_-EP3 pathway plays crucial roles in the lymphatic specification from venous to lymphatic endothelial cells through the upregulation of *sox18* and *nr2f2*. These data strongly suggest a novel function of COX1-PGE_2_-EP3 pathway in the formation of the lymphatic system during early development.

## Materials and Methods

### Materials

The following materials were obtained from the sources indicated; PGE_2_, sulprostone, and SC-560 from Cayman Chemical (Ann Arbor, MI), indomethacin from Sigma-Aldrich (St. Louis, MO), Calcium 5 Assay Kit from Molecular Devices (Sunnyvale, CA), LightCycler 480 SYBR Green I Master and blocking reagent from Roche Diagnostics (Mannheim, Germany). The EP-specific agonists, ONO-AE1-259 (EP2) and ONO-AE1-329 (EP4), were generous gifts from Ono Pharmaceutical Co. (Osaka, Japan). All other chemicals were commercial products of reagent grade.

### Zebrafish line and maintenance

A wild-type zebrafish strain was obtained from National BioResource Project Zebrafish (RIKEN, Japan). The transgenic zebrafish line Tg(fli1a:egfp) has been described previously^27,32^, and was used to monitor lymphatic development. Zebrafish were maintained at 28.5 °C under a 14 h-light/10 h-dark cycle. Embryos were maintained in 1/3 ringer solution buffer at 28.5 °C. All experimental protocols were approved by Kumamoto University (24-060, F29-195). All experiments with zebrafish were performed in accordance with the guidelines of Kumamoto University.

### Morpholino antisense oligos

EP3 MO1 (targeting the splice site between exon 1 and intron 1), EP3 MO2 (targeting the splice site between intron 1 and exon 2), and Cont MO were purchased from Gene Tools, LLC (Philomath, OR). Each MO (10 ng) was injected into the yolk of 1–2 cell stage embryos. The sequence of each MO is shown in Supplementary Table S1.

### RNA extraction and RT-qPCR

Total RNA was extracted from zebrafish embryos at the indicated stages using Sepasol RNA I Super G (Nacalai Tesque, Kyoto, Japan), and was subjected to RT with PrimeScript RT Master Mix (Takara Bio, Shiga, Japan). Synthesized cDNA was subjected to qPCR using a LightCycler (Roche Applied Science, Penzberg, Germany) and Fast Start DNA Master SYBR Green I according to the manufacturer’s instructions. Crossing point values were acquired by the second derivative maximum method. The expression level of each gene was quantified using external standardized dilutions. Relative expression levels among samples were normalized by the value of *gapdh*. Sequences of the used primers are shown in Supplementary Table S2. The specificity of qPCR was confirmed by the lengths and melting temperatures of the amplified products.

### Whole-mount *in situ* hybridization

Total RNA was isolated from zebrafish embryos, and cDNA was synthesized using SuperScript III (Invitrogen, San Diego, CA) and oligo (dT) primers. The coding sequence of each gene (*lyve1b*, *nr2f2*, *hey2*, and *flt4*,) were amplified from the cDNA by PCR and cloned into the pTA2 vector (Toyobo, Osaka, Japan). Primer sequences used in the PCR are shown in Supplementary Table S3. Cloning of the coding sequences of *gata1a*, the EP3 receptor, COX1, COX2a, and COX2b was performed as previously described^20,41^. These plasmids were linearized by restriction enzymes. Digoxigenin (DIG)-labeled anti-sense RNA was transcribed from each linearized vector by *in vitro* transcription using DIG mix and transcription buffer (Roche Diagnostics). These RNA was purified by ethanol precipitation, dissolved in hybridization buffer (50% formamide, 5× SSC, 5 mM ethylenediaminetetraacetic acid, 0.1% Tween-20, and 1 mg/mL torula RNA), and used as hybridization probes. WISH was performed as previously described^41^. After fixation by 4% paraformaldehyde, zebrafish embryos at 52 hpf were incubated in 3% hydrogen peroxide solution to remove dark pigments. Embryos stained with WISH were embedded in OCT Compound (Sakura, Tokyo, Japan) and transverse sections (10–20 μm) were prepared using a cryostat (Leica Microsystems, Wetzlar, Germany). Images were taken with a fluorescence microscope (BZ-X700; KEYENCE, Osaka, Japan) and were processed using the attached software or Adobe Photoshop (Adobe, San Jose, CA).

### Generation of EP3 receptor-mutant zebrafish

TALEN plasmids were constructed using a two-step assembly system, as described previously^42^. Briefly, six or fewer TAL effector repeat domains were ligated into the pFUS vector^43^. Subsequently, each pFUS vector and last TAL effector repeat were ligated into the pCS2 vector as a TALEN plasmid^44^. The TALEN plasmids were linearized by Not I digestion, and TALEN mRNA was transcribed using the mMESSAGE mMACHINE SP6 kit (Life Technologies, Gaithersburg, MD) and purified using the RNeasy Mini Kit (QIAGEN, Hilden, Germany). Forward and reverse TALEN mRNA (400 pg each) was simultaneously injected into zebrafish blastomeres at the one-cell stage. To detect genome modification in zebrafish, we utilized the heteroduplex mobility assay as reported previously^45^. The sequences of the primers used in the heteroduplex mobility assay are shown in Supplementary Table S4. To check the functional ability of the TALEN-induced EP3 receptor mutant, the Ca^2+^ mobilization assay was performed using the FLIPR Calcium 5 Assay Kit (Molecular Devices, Sunnyvale, CA) according to the manufacturer’s instructions. HeLa cells were grown in modified Eagle’s medium (Sigma-Aldrich) supplemented with 10% fetal bovine serum at 37 °C in a fully humidified CO_2_ atmosphere. Wild type or mutant EP3 receptor constructs were transfected into HeLa cells using FuGENE HD (Promega, Madison, WI) according to the manufacturer’s instructions. After 24 h, transfected cells were labeled with calcium 5 loading buffer for 1 h, and stimulated with sulprostone. Fluorescence (excitation, 485 nm; emission, 515 nm) was monitored for 90 seconds on FlexStation III (Molecular Devices) and the area under curve (AUC) was evaluated as induced intracellular Ca^2+^ mobilization.

### Statistical analysis

Data are shown as the mean ± SEM. Comparison of two groups was analyzed by the Student’s *t*-test. For comparison of more than two groups with comparable variances, one-way ANOVA was performed, and the Tukey’s test was subsequently used to evaluate the pairwise group difference. *P*-values less than 0.05 were considered to indicate significant differences.

## Supplementary information

Appendix
