# Supplementary material for: Essential role of prostaglandin E2 and the EP3 receptor in lymphatic vessel development during zebrafish embryogenesis
Source: Sci Rep. 2019 May 21;9:7650. doi: 10.1038/s41598-019-44095-5 (PMC6529442; doi:10.1038/s41598-019-44095-5)
Supplement: Supplementary file 1 — Appendix [file 41598_2019_44095_MOESM1_ESM.pdf]

Supplementary Information:

Essential role of prostaglandin E_2 and the EP3 receptor in lymphatic vessel development during zebrafish embryogenesis

Ryo Iwasaki[1], Kyoshiro Tsuge[1], Koichiro Kishimoto[1], Yuta Hayashi[1], Takuya Iwaana[1], Hirofumi Hohjoh[1], Tomoaki Inazumi[1,2], Atsuo Kawahara[3], Soken Tsuchiya[1,2,#], and Yukihiko Sugimoto[1,2,#]

[1]Department of Pharmaceutical Biochemistry, Graduate School of Pharmaceutical Sciences, Kumamoto University, Chuo-ku, Kumamoto 862-0973, Japan
[2]Japan Agency for Medical Research and Development-Core Research for Evolutional Science and Technology (AMED-CREST), Chiyoda-ku, Tokyo 100-0004, Japan
[3]Laboratory for Developmental Biology, Center for Medical Education and Sciences, Graduate School of Medical Science, University of Yamanashi, Shimokato 1110, Chuo, Yamanashi, 409-3898, Japan

[#]Correspondence authors

Supplementary figure legend

Supplementary Table S1 to S4
Supplementary Figure S1

Supplementary figure legend

Supplementary Figure S1. Target sites and effect of EP3 receptor MOs. (A) Schematic representation of MO targeting sites in the EP3 receptor gene. Grey boxes represent the transmembrane regions. (B) Embryos were injected with Cont MO, EP3 MO1, or MO2. Expression levels of EP3 receptor mRNA were quantified by RT-qPCR in morphants at 24 hpf. The values are shown relative to the value obtained with Cont MO. $^{**}P < 0.01$ vs Cont MO. Each value represents the mean ± SEM (N = 5-10).

Supplementary Table S1. Sequence of MOs targeting the zebrafish EP3 receptor.

Name	Sequence (5′ to 3′)
Control MO	cctcttacctcagttacaatttata
EP3 MO1	agctgataggatacataccagtaga
EP3 MO2	acctgcaaaatgggaggagacataa

Supplementary Table S2. Sequences of primers used for RT-qPCR analysis.

Gene symbol	Forward primer (5′ to 3′)	Reverse primer (5′ to 3′)
ptger3	ttattcagttgatgggcattatgt	aattacagtccttctgcaattcct
lyve1b	tgccatcacagccaaagaggc	gctcctcaacccaaccaaacctgc
vegfc	gcttcagtgcaggaacatca	atgattgagtgctgctgtcg
flt4	tgactcgggttattaccgctgctt	agatggtctccatgtcgttgtctc
sox18	tccttggacgctgtggaccaac	tcaaagcgctgctttcctcgc
nr2f2	acacagtcaaccccgacgaacc	tttgtccccgcaaaccacgc
apln	tgaagatcttgacgctggtg	gcaaaggagtcctcatgctt
ccbe1	gggttgtttgcacctgctat	gcatgtcttcccatcatcct
wnt5b	aagacgggcatcaaagagtg	cgtaaaagctgtttctcggc
bmp2b	ccagcagagcaaacacgata	tgtggaagccactcgtactg
gapdh	gatacacggagcaccaggtt	gccatcaggtcacatacacg

Supplementary Table S3. Sequence of primers used for cloning.

Gene symbol	Forward primer (5′ to 3′)	Reverse primer (5′ to 3′)
lyve1b	tttagaaggttttggtggcatgtt	aaaacagcatatcttaggaagagtcaga
nr2f2	tgatagatatggcaatggtagtgtgg	ctcttgtaacagccgtttccttctac
hey2	aatgaagtttgagacctccattcg	aatagccgctgctttcctttatgt
flt4	gactgcaagagtctgcacca	cccagagcagaactccaaaa

Supplementary Table S4. Sequence of primers used for the heteroduplex mobility assay of the EP3 receptor.

Gene symbol	Forward primer (5′ to 3′)	Reverse primer (5′ to 3′)
ptger3	gctgcgggtcggtgtccgtgttt	gccaatgaaccgatgcaaagcaga

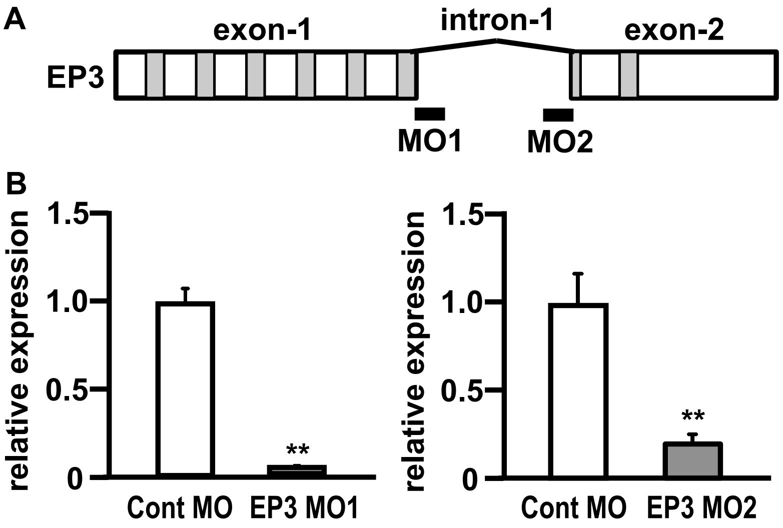
A
exon-1
intron-1
exon-2
EP3
MO1
MO2
B
relative expression
1.5
1.0
0.5
0
Cont MO
EP3 MO1
**
relative expression
1.5
1.0
0.5
0
Cont MO
EP3 MO2
**
